# Prenatal hepatitis B screening and associated factors in a high prevalence district of Lira, northern Uganda: a community based cross sectional study

**DOI:** 10.1186/s12889-019-7344-6

**Published:** 2019-07-26

**Authors:** Paul Semakula Katamba, David Mukunya, Doris Kwesiga, Victoria Nankabirwa

**Affiliations:** College of Health Sciences, School of Public Health, Makerere University, Kampala, Uganda

**Keywords:** Prenatal, Hepatitis B screening, Target journal: BMC public Health

## Abstract

**Background:**

Chronic hepatitis B Virus (HBV) infection affects 80–100 million people in sub-Saharan Africa and accounts for an estimated 650,000 deaths annually. The prevalence of active hepatitis B virus infection among women aged 15–64 in mid-Northern Uganda is about 5%. Lira district is among the high prevalence areas where government embarked on mass HBV screening since 2015 as a gateway for access to prevention, treatment services, and an effective response to the hepatitis B epidemic. The current proportion of pregnant women screened and the factors associated with prenatal HBVscreening in Lira are not known despite the fact that women contribute largely to both vertical and horizontal transmission of HBV. This study aimed at determining the proportion of pregnant women screened for HBV and factors associated with prenatal HBV screening in Lira district.

**Methods:**

This was a community based cross sectional study conducted among 423 pregnant women in the sub counties of Aromo and Agweng in Lira district. Data were collected using open data kit and analysed using STATA version 14. The outcome variable was prenatal HBV screening while predictor variables were community, individual and health facility factors associated with HBV screening. Multivariable logistic regression was used to determine factors associated with prenatal HBV screening.

**Results:**

Thirty five women (8.3%) had been screened for HBV during the current pregnancy. Factors associated with prenatal HBV screening in Lira included perceived risk (Adjusted Odds Ratio (AOR) 3.78, 95% CI 1.01–6.14), respondent’s age (AOR = 3.98, 95% CI 1.39–5.09), husband/partner’s education (AOR = 3.34, 95% CI 1.10–5.12) and past failure to access to HBV screening services at government health facilities (AOR = 6.44, 95% CI 2.10–8.02).

**Conclusion:**

The level of HBV screening among pregnant women in Lira was low and is mainly associated with perceived risk, age, access to HBV screening services and spousal education level. More effort is needed in creating mass awareness on the need and importance of HBV screening most especially among pregnant women.

## Background

Globally, about 248 million people are living with chronic Hepatitis B Virus (HBV) infection and the burden of HBV remains disproportionately high in low and middle-income countries (LMICs), particularly in Asia and Africa [1]. Chronic HBV infection affects 80–100 million people in sub-Saharan Africa and is a major cause of mortality from decompensated cirrhosis and hepatocellular carcinoma [2]. According to the Uganda population-based HIV impact assessment survey 2016/2017, hepatitis B prevalence among women aged 15–64 is 3% in Uganda and about 5% in the mid-North region where Lira district is located [3].

Routine Hepatitis B surface antigen (HBsAg) serological testing of all pregnant women in antenatal clinics and linkage to prevention, care and treatment services is the recommended standard way of controlling and reducing neonatal HBV transmission in settings where HBsAg seroprevalence in the general population exceeds 2% [1]. To this effect, the Ministry of Health in Uganda embarked on mass screening in addition to opening up specialized HBV clinics in several high prevalence areas including Lira district [4]. Information accessed from the District Health Information System (DHIS) shows that in Lira district, 13609 people were screened for HBV out of whom 8.5% (1150) tested positive between June 2017 and June 2018.

Vertical transmission is the main route by which HBV infection is perpetuated [5]. Most people with chronic HBV acquire the infection during the perinatal period and early childhood [6]. HBV mother-to-child transmission occurs in 2–30% of infants born to HBV-infected mothers both vertically and horizontally [7]. The risk of becoming a chronic hepatitis B infection carrier is 95% for infections acquired during the perinatal period compared with 5% for those acquired during adulthood [5]. Interrupting early transmission through maternal HBV screening and vaccination is key to breaking the cycle of ongoing HBV infection [6].

In settings where the prevalence of HBV infection among women of child-bearing age is greater than 5%, national policies for routine vaccination at birth are a prerequisite for control and prevention [8]. It is recommended that all pregnant women should be screened routinely for HBsAg in every prenatal visit whether they have been previously vaccinated or screened [9]. Universal screening for HBV in pregnant women has had a major impact in decreasing the risk of neonatal infection by identifying HBV-infected mothers whose babies can benefit from HBV vaccinations in combination with prophylaxis with hepatitis B immunoglobulins (HBIgs) [10].

In Uganda, national mass screening and vaccination programme was launched in 2015 and over 200 point of care machines (Selexon) that can be used to screen for hepatitis B were acquired [4]. The machines have since been distributed to major public hospitals and health centers in areas with high prevalence, including Lira district [4]. While a lot is being done, there is a need to assess the health seeking behavior of women meant to benefit from such programs. Identification of characteristics of women, their perceptions of own risk and barriers to accessing existing screening services can provide important information for shaping screening services as developing countries prepare to adopt emerging and more affordable technologies [11]. This study aimed at estimating the proportion of pregnant women screened for HBV and factors associated with prenatal HBV screening in Lira district.

## Methods

### Study site

We carried out the study in Agweng and Aromo sub counties, Lira district. These sub-counties have the highest rates of maternal and child mortality in Lira district, estimated at over 400 per 100,000 live births and 85 per 1000 live births respectively [12]. Lira District is located in Lango sub-region in Northern Uganda and is bordered by the districts of Pader and Otuke in the North and North East, Alebtong in the East, Dokolo in the South and Apac in the West. It is 375kms from Kampala via Karuma- Kamdini. The total population in Lira district is about 403,100. Most of the inhabitants are subsistence peasant farmers.

### Study design and sampling procedure

This was a community-based cross sectional study nested in a cluster-randomized trial that considered the same study population (pregnant women). The cluster randomized trial was on the effectiveness of an integrated intervention consisting of pregnancy buddies, mobile phone messages, and mama kits in increasing facility-based births. Sample size estimation was done using the Kish Leslie formula for cross sectional studies [13] and a 10% adjustment for non-response was made to come up with 423 respondents.

$$ \frac{N={Z_{\alpha}}^2P\ \left(1-P\right)}{\delta^2} $$

Where N = sample size estimate of pregnant women.

P = assumed true population prevalence of Hepatitis B screening services (50%).

Z_α_ = Standard normal deviate at 95% confidence interval corresponding to 1.96.

δ = Absolute error between the estimated and true population prevalence of Hepatitis B screening, (5%) at 95% CI.

Consecutive sampling method was used whereby every woman known to be pregnant within every village in the two sub-counties was approached and those who were eligible and consented to participate in the study were included until a sufficient sample size was accrued.

Though not a probability sampling method, it allows one to select all the accessible population in an area during the study period. This method is recommended for RCTs including the one in which our study was nested.

### Study variables

The outcome variable was self reported prenatal hepatitis B screening. A pregnant woman was included if she self-reported to have been screened for Hepatitis B since conception of the current pregnancy. Independent variables were community, individual and health facility factors that affected prenatal Hepatitis B screening. Community factors included cultural beliefs and practices, stigma, community mobilisation and sensitisation. Individual factors included; formal education level, gender, age, marital status, attitude towards the services, knowledge and awareness about hepatitis B infection, HBV transmission, HBV screening perceived risk and complications. Health facility factors included; health worker attitude, availability of skilled health workers, convenience of obtaining care, cost, and distance.

A pregnant woman was eligible for the study if she was in the last trimester of her pregnancy (i.e., based on self-reported information using dates for the last normal menstrual period) and was a resident in one of the two sub counties. Pregnant women with psychiatric ailments that prevented them from providing an informed consent were excluded.

### Data collection

We employed quantitative data collection methods. Data were collected electronically using interviewer administered structured, standardized, pre-coded and pre-tested questionnaires. The questionnaires were prepared in English and translated to Lango and then back-translated to English to ensure consistency of the tool. Data were uploaded into ODK software on android mobile phone devices that were configured to have an instant check for validity and could not allow certain types of erroneous responses to be entered. Range and consistency checks were also incorporated in the data collection system to ensure completeness.

The original study recruited pregnancy monitors in every village of the study area. These were elected by the community in a public meeting. Their role was to identify all pregnant women in the area, and inform the study team. In order to ensure that all pregnant women had been enrolled, the study also employed village health team leaders to conduct a census of all pregnant women in the study area. At the end of each field day, data would be uploaded into a secure database that was encrypted and password protected to preserve participant confidentiality. Research assistants that fluently spoke Lango were recruited from the study area and trained on electronic data collection.

### Data analysis

Data were cleaned using MS EXCEL and exported to STATA version 14 for analysis. Frequencies and percentages were obtained for all categorical variables and means (Standard Deviation) and medians (Inter Quartile Range) were generated for continuous variables. The number of pregnant women that had been screened for HBV during the current pregnancy was expressed as a proportion of the total sample size. Bivariable logistic regression was used to examine the crude association between prenatal HBV screening and the predictor variables. Variables that had *p* < 0.25 [14] at bivariable analysis, those backed by literature were considered in a stepwise and logical way for the multivariable models aimed at determining independent factors associated with the outcome. The most parsimonious model was tested for goodness of fit using the Archer-Lemeshow goodness-of-fit test (l fit test). Considering the null hypothesis that the model was a good fit, the model was found to have a *p* > 0.05 using the 95% level of significance, which implied that it was a good fit.

## Results

### Baseline characteristics of respondents

The study was carried out among 423 respondents. The mean age (SD) was 24 (6.22) ranging from 14 years to 42 years. They had a mean of 5.1 years of formal education ranging from 0 to 18 years. Mean parity was 3, ranging from 0 to 10*(*Table 1*).*

**Table 1:** Baseline characteristics of respondents

Variable	N (%)
Age (mean = 24)	
≤ 25 years	251 (59.2)
> 25 years	173 (48.8)
Formal Education (mean = 5.1)	
≤ 7 years	375 (88.6)
> 7 years	48 (11.4)
Tribe	
Langi	404 (95.5)
Other	19 (4.5)
Marital status	
Married	379 (89.6)
Unmarried	44 (10.4)
Previous pregnancies had	
0	114 (27.0)
1–3	185 (43.7)
≥ 4	124 (29.3)
Parity(*n* = 310)	
0	15 (4.8)
1–2	131 (42.3)
≥ 3	164 (52.9)
Household income provider	
Husband/Partner	371 (87.2)
Myself	20 (5.0)
Others	33 (7.8)
Earning own money(*n* = 402)	
No	296 (73.6)
Yes	106 (26.4)
Household head	
Husband/Partner	389 (92.0)
Myself	16 (3.8)
Other	18 (4.2)
Husband/Partner’s occupation(*n* = 380)	
Farming	298 (78.4)
other	82 (21.6)
Attending Antenatal clinic	
No	143 (34.0)
Yes	280 (66.0)

Most of the respondents 389 (92.0%) were from households headed by men (husbands/partners).

Three hundred seventy nine (89.6%) participants were either married or cohabiting. Majority of the respondents 404(95.5%) were Langi. Of all the pregnant women interviewed, 114(27.0%) were pregnant for the first time and 280 (66%) had started attending antenatal care clinics by the time of the study *(*Table 1*).*

### Prenatal HBV screening

Thirty five (8.3%) respondents had been screened during the current pregnancy at the time of the study. Majority of the pregnant women interviewed (*n* = 351, 83.0%) were aware (knew of /had heard about) hepatitis B disease, 25.1% (106) knew how HBV is transmitted, 42.8%(181) did not think that they were at risk of HBV infection while 15.6%(66) were aware about HBV screening. One hundred twenty one (28.6%) knew where screening services were offered in Lira district (Table 2)*.*

**Table 2:** Factors associated with prenatal hepatitis B screening in Lira District

Variable	Screened since conception		Unadjusted Odds Ratio	Adjusted odds ratio (AOR)
	No n (%)	Yes n (%)		
Age				
< 25	90 (60)	14 (41.2)	1	1
≥ 25	60 (40)	20 (58.8)	2.14 (1.01–4.57)	3.98 (1.39–5.09)*
Formal education				
≤ 7 years	124(82.7)	30 (85.7)	1	
> 7 years	26 (17.3)	5 (14.3)	0.79 (0.28–2.24)	
Marital status				
Married	134(89.3)	31 (88.6)	1	
Unmarried	16 (10.7)	4 (11.4)	1.08 (0.34–3.46)	
Pregnancy had before				
0	47(31.33)	6 (17.14)	1	
1–3	62(41.33)	18(51.43)	2.27 (0.84–6.17)	
>3	41(27.33)	11(31.43)	2.10 (0.71–6.18)	
Husband’s education level				
< 7 years	78 (58.2)	15 (48.4)	1	1
≥ 7	56 (41.8)	16 (51.6)	1.49 (0.68–3.25)	3.25 (1.04–5.12)*
Earning own money				
No	99 (68.3)	22 (71.0)	1	
Yes	46 (31.7)	9 (29.0)	0.88 (0.38–2.06)	
Knows how HBV is transmitted				
No	89 (59.3)	18 (51.4)	1	
Yes	61 (40.7)	17 (48.6)	1.38 (0.66–2.88)	
Aware of HBV screening				
No	20 (13.3)	1 (2.9)	1	
Yes	130(86.7)	34 (97.1)	5.23 (0.68–40.37)	
Knows where to screen from				
No	68 (51.5)	13 (38.2)	1	
Yes	64 (48.5)	21 (61.8)	1.72 (0.79–3.71)	
Perceived risk				
I don’t know	92 (61.3)	20 (57.2)	1	1
No	37 (24.7)	6 (17.1)	0.75 (0.27–2.00)	1.60 (0.39–6.56)
Yes	21 (14.0)	9 (25.7)	1.97 (079–4.94)	4.66 (1.20–6.14)*
Stigma in community				
No	99 (66.0)	27 (77.1)	1	
Yes	14 (9.3)	3 (8.6)	0.79 (0.21–2.93)	
I don’t know	37 (24.7)	5 (14.3)	049(.018–1.38)	
Husband/partner’s approval				
No	52 (34.7)	13 (37.1)	1	
Yes	98 (65.3)	22 (62.9)	0.90 (0.42–1.93)	
Health worker attitude				
Poor	10 (7.6)	1 (3.2)	1	
Fair	61 (46.2)	19 (61.3)	3.11 (037–25.93)	
Good	61 (46.2)	11 (35.5)	1.80 (021–15.53)	
Readily accessed screening services				
Yes	85 (64.4)	16 (51.6)	1	1
No	47 (35.6)	15 (48.4)	1.69 (0.77–3.73)	6.44 (2.10–0.02)**
Campaign against screening				
No	130(89.0)	23 (85.2)		
Yes	16 (11.0)	4 (14.8)	1.41 (0.43–4.61)	

Note: *(p < 0.05), ** (p < 0.01)

*Variables with p-value < 0.05 are significantly associated with HBV screening*

### Factors associated with prenatal HBV screening

Age of the respondent was independently associated with prenatal HBV screening in Lira district. The odds of prenatal HBV screening among women aged above 25 years were 4 times those among pregnant women aged below 25 years (AOR 3.98, 95% CI 1.39–5.09) *(*Table 2*).*

Perceived risk of HBV infection was independently associated with prenatal HBV screening. The odds of prenatal HBV screening among women who perceived themselves as being at risk of HBV infection were 4 times those among women who didn’t (AOR 4.66 95% CI 1.20–6.14) *(*Table 2*).* Husband/partner’s education level was independently associated with prenatal HBV screening. The odds of prenatal HBV screening among women whose husbands/partners had attained more than 7 years of formal education were 3 times those among women whose husbands/partners had attained less than 7 years of formal education (AOR 3.25, 95% CI 1.10–5.12) *(*Table 2*).*

Access to HBV screening services at the health facilities was independently associated with prenatal HBV screening. The odds of prenatal HBV screening among women who readily accessed the services at the nearest health facilities were 6.44 times those among women who didn’t readily access the services (AOR 6.44, 95% CI 2.10–8.02) *(*Table 2*)*.

## Discussion

We found that out of the 423 pregnant women interviewed, 8.3% had been screened for HBV during their current pregnancy. Being older, having more than 7 years of education and easy access to HBV screening services were associated with prenatal HBV screening among pregnant women in Lira district.

The low level of HBV screening could be attributed to insufficient sensitization and awareness campaigns with regard to HBV screening in Uganda. Hepatitis B has generally not attracted enough government attention compared to other communicable diseases like HIV/AIDS and tuberculosis. The 2016 UDHS results show that in the northern region where Lira district is located, 97.1% of women who gave birth in the 5 years preceding the survey received antenatal care (ANC) from a skilled provider at least once for their last birth and 55.9% of pregnant women had the recommended four or more ANC. Even when STD/HIV/AIDS control is one of the pillars of safe motherhood and HIV testing is mandatory for all pregnant women seeking ANC services [4], HBV testing isn’t. Analysis of national survey data in Brazil showed that 29% of the respondents reported to have been screened for HBV [15]. A facility based cross sectional studies in Nigeria and Uganda showed that HBV screening among health workers was also low at 22 and 44.3% respectively [16, 17]. Both studies however did not specifically target pregnant women per se.

We found that age was positively associated with prenatal HBV screening. The result is in agreement with findings from other studies which indicated that the increase in age of a pregnant mother increases the use of health services [18, 19]. This could be because of the fact that women aged above 25 years are more mature and have probably been exposed to a lot more sensitization on the importance of good prenatal health for both the mother and the unborn baby. Studies conducted in Ghana and Kenya on utilisation of maternal healthcare services including HBV screening reveal that antenatal care utilisation reduced with increase in age of expectant mothers [20, 21]. Though both studies were cross sectional, they used larger sample size from national survey data which may be the reason for the different results from our study.

Findings from our study showed significant influence of higher education levels of men on the use of maternal health services and were consistent with a study conducted in India [22]. Better educated men are more aware and concerned about their partners’ health, know more about availability of maternal health care services and use this awareness and information in supporting the women’s access to the health services. Educated men tend to oppose some of the negative cultural beliefs that may be deeply rooted in rural communities such as Lira as regards decision making on when a women has to visit a health facility or not. Unlike in the cross sectional studies in Nigeria [23] and Ghana [24], this study didn’t show a significant association between women’s education and utilisation of HBV services. Though these involved larger sample sizes and different sampling procedures, they did not target pregnant women specifically. Thus this may have caused the difference in findings.

The study showed that women who perceived themselves to be at risk of HBV infection were more likely to utilize HBV screening services compared to those who perceived themselves not at risk of HBV infection. A cross-sectional survey by Greenaway [25] revealed that women with a higher underestimation of perceived risk for infection would be less likely to screen for a given condition. From the health belief model, individuals who perceive that they are susceptible to a particular health problem engage in behaviors to reduce their risk of developing the health problem while individuals with low perceived susceptibility may deny that they are at risk for contracting a particular illness [26]. Others may acknowledge the possibility that they could develop the illness, but believe it is unlikely [26]. However, some women who didn’t perceive themselves at risk of HBV infection were screened for HBV. This could probably be due to peer influence or having a husband/partner who appreciated and demanded their partners to be screened for HBV.

Though not significantly associated with HBV screening, health worker attitude showed a strong association that required further investigation. Findings from this study showed that women who said that the attitude of health workers was good were three times more likely to use the screening services offered than the women who said that health workers attitude was poor. Though not statistically significant in this study, the result is too large for only the assumption of chance to be considered. The conscious effort of health workers to behave favourably towards clients suggests that their attitudes and behaviours were meant to create the impression that they related to and treated clients well. Majority of the respondents continue to seek services from certain hospitals because health workers treat them well [27].

However, our study used consecutive sampling method and was nested in a cluster randomized trial in the sub counties with the worst maternal and child health indicators thus the results could possibly be an underestimate of the reality and therefore may not be generalizable to the whole of Lira district.

Social desirability bias could have been introduced since we obtained data from the respondents self reports which information we did not validate with an objective source such as health facility cards.

The study relied on cross-sectional data, so we could not draw conclusions about causality. There is a possibility that the relationships found in this study are due to the influence of other variables that were not studied, reverse causation and or measurement error.

## Conclusion

The level of prenatal HBV screening services among pregnant women was low (8%) in such a high prevalence area in Uganda. Factors positively associated with prenatal HBV screening in Lira district were; age of respondents, husband/partner’s education, perceived risk and access to HBV screening services at the nearest health facility. There is need for government to consider targeted screening especially among women in the area coupled with vaccination and linkage to appropriate care. Government should ensure more sensitization and awareness campaigns in the communities about the need and importance of HBV screening with more emphasis on younger women and also consider availing HBV screening services in all health facilities where antenatal care services are provided. Implementation of a mandatory policy on routine prenatal HBV screening should be considered, adhered to and sustained.

## Data Availability

The datasets used and/or analysed during for our study are available from the corresponding author on reasonable request.

## Abbreviations

HBIG: Hepatitis B Immunoglobulin
HBsAg: Hepatitis B Surface Antigen
HBV: Hepatitis B Virus
LDLG: Lira District Local Government
LMICs: Low and Middle-Income Countries
MOH: Ministry Of Health
WHO: World Health Organization
